# A Multi-Site Survey Study on the Association Between the COVID-19 Pandemic and United States Anesthesiology Residents’ Mental Health

**DOI:** 10.7759/cureus.34782

**Published:** 2023-02-08

**Authors:** Fei Chen, Robert Isaak, Farzana Afroze, Teresa A Mulaikal, Lauren K Licatino, Beth Ladlie, Ankit Jain, Chelsea Willie, Emily Bairde, Blair H Hayes, Tekuila Carter, Lara Zisblatt, Carol Diachun, Timothy W Martin, Julie M Marshall, Julie Huffmyer, Anna K Hindle, David L Stahl, Yutong Liu, Susan M Martinelli

**Affiliations:** 1 Department of Anesthesiology, University of North Carolina School of Medicine, Chapel Hill, USA; 2 Department of Anesthesiology, Albany Medical Center, Albany, USA; 3 Department of Anesthesiology, Columbia University, New York City, USA; 4 Department of Anesthesiology and Perioperative Medicine, Mayo Clinic, Rochester, USA; 5 Department of Anesthesiology and Perioperative Medicine, Mayo Clinic, Jacksonville, USA; 6 Department of Anesthesiology and Perioperative Medicine, Medical College of Georgia, Augusta University, Augusta, USA; 7 Department of Anesthesiology and Pediatrics, Medical College of Wisconsin, Milwaukee, USA; 8 Department of Anesthesiology, Oregon Health & Science University, Portland, USA; 9 Department of Anesthesiology, The Ohio State University, Columbus, USA; 10 Department of Anesthesiology and Perioperative Medicine, University of Alabama at Birmingham, Birmingham, USA; 11 Department of Anesthesiology, University of Michigan, Ann Arbor, USA; 12 Department of Anesthesiology, University of Florida College of Medicine, Jacksonville, USA; 13 Department of Anesthesiology, University of Florida, Gainesville, USA; 14 Department of Anesthesiology, University of Missouri, Columbia, USA; 15 Department of Anesthesiology, University of Virginia School of Medicine, Charlottesville, USA; 16 Department of Anesthesiology, The George Washington University, Washington, D.C., USA; 17 Department of Biostatistics, University of North Carolina, Chapel Hill, USA; 18 Department of Anesthesiology, University of North Carolina School of Medicine, Chapel HIll, USA

**Keywords:** year 2 students, impact of covid-19, u.s. medical residency, covid-19, mental health, post grad medical education, anesthesia, resident well-being

## Abstract

Background: At the onset of the coronavirus disease 2019 (COVID-19) pandemic, anesthesiology residency programs were impacted differently due to various factors such as the local severity of COVID-19, exposure to patient suffering, and inability to complete rotations. We sought to investigate the impact of local-level pandemic severity on the well-being of anesthesiology residents.

Methods: This multi-site study surveyed postgraduate year two residents from 15 United States (US) anesthesiology programs using the Perceived Stress Scale, Mini-Z, Patient Health Questionnaire-9,WHO-5 Well-Being Index,and the Multidimensional Scale of Perceived Social Support before the pandemic (baseline survey) and during the first COVID-19 surge (post survey).

Results: A total of 144 (65%) residents responded to the initial baseline survey; 73 (33%) responded to the post survey, and 49 (22%) completed both surveys. There was not a statistically significant difference in any well-being outcomes of participants between the surveys, nor was there a significant difference based on the severity of COVID-19 impact at the program’s hospital. Male participants had higher perceived stress scores (β = 4.05, 95%CI: 0.42, 7.67, P = 0.03) and lower social support from family (β = -6.57, 95%CI: -11.64, -1.51, P = 0.01) at the post survey compared to female participants after controlling for baseline scores. Additionally, married participants or those with domestic partners reported higher perceived social support in the post survey (β = 5.79, 95%CI: -0.65, 12.23, P = 0.03).

Conclusion: The local COVID-19 severity at a residency program did not disproportionately impact well-being scores among anesthesiology residents. Those most vulnerable to diminished well-being appeared to be male and single participants. As a result, targeted well-being interventions, including those aiming to increase social support, to higher-risk resident groups may be indicated. Future work is needed to assess the longstanding COVID-19 pandemic impacts on resident well-being.

## Introduction

When the coronavirus disease 2019 (COVID-19) pandemic began affecting the United States (US) in March 2020, graduate medical education was significantly impacted, structurally and functionally. In several areas of the country with high COVID-19 disease burdens, some residents were redeployed to diverse and unfamiliar environments throughout hospital systems to provide clinical workforce support. Some hospital systems were so overwhelmed by the pandemic that operating rooms (ORs) were repurposed into intensive care units and resident anaesthesiologists served as bedside critical care providers [1,2]. During this time of uncertainty and change, many residents were fraught with concern about their own personal health and the health of their loved ones. Additionally, because of the disease penetrance, many residents were exposed to an atypical high degree of patient, caregiver, and family suffering. Residents who worked in areas with a low COVID-19 patient burden were also affected, but in distinctly different ways than those in areas of high COVID-19 severity. For instance, as hospital systems electively decreased surgical volumes to conserve resources, many residents failed to receive the necessary experiences to complete rotations and case-log requirements [3]. The structure of education and programming within anesthesiology training programs shifted as well, with reduced simulation programming and a transition of didactics to virtual platforms [4]. Importantly, studies from multiple countries reported that residents also experienced non-work-related stresses, including the inability to socialize with friends and family members, restrictions on travel, and challenges with obtaining child care [5-10].

The impact of the COVID-19 pandemic on anesthesiology residents has been previously described [10]. However, many of the reported studies were selective overviews or cross-sectional surveys, which cannot offer a longitudinal assessment of shifts in trainees’ mental health status [10,11]. This multi-site study aimed to assess the impact of the COVID-19 pandemic on the well-being of anesthesiology residents in the US. We hypothesized that: (i) residents would report lower levels of well-being during the first few months of the COVID-19 outbreak as compared to their pre-pandemic baseline across the institutions, and that (ii) residents at hospitals with higher levels of COVID-19 case burden would report lower levels of well-being compared to those at programs of less severe COVID-19 status. Additionally, we explored the association between several demographic variables (e.g., gender, marriage status) and well-being during the initial outbreak of COVID-19.

## Materials and methods

This multi-site study was approved by the Institutional Review Board of the University of North Carolina at Chapel Hill, North Carolina, United States (Approval number: 18-3299).

Electronic mental health surveys were distributed to 222 postgraduate year two (PGY-2) clinical anesthesiology (CA1) residents at 15 US-based anesthesiology residency programs in September and October of 2019 (Baseline survey) and subsequently in March and April of 2020 (Post survey). Mental health measures used in the surveys included: the Perceived Stress Scale, Mini-Z, Patient Health Questionnaire-9, WHO-5 Well-Being Index, and the Multidimensional Scale of Perceived Social Support [12-16]. These surveys were distributed as a component of a larger study investigating the well-being of anesthesiology residents over an extended time period. Due to the post survey coinciding with the early stage of COVID-19, the data was utilized to explore the effect of the early stages of the COVID-19 pandemic on the well-being of anesthesiology residents. Participation in the study was voluntary, and participants were allowed to withdraw at any time. The surveys were distributed and managed using the REDCap electronic data capture tool hosted at the University of North Carolina at Chapel Hill [17].

In the fall of 2020, we retrospectively contacted the primary investigators of the participating sites, inviting them to complete a Qualtrics survey (Qualtrics, Seattle, Washington, United States) reporting their program’s highest Accreditation Council for Graduate Medical Education (ACGME) COVID-19 stage designation (Stage one = business as usual, Stage two = increased but manageable clinical demand, Stage three = clinical demand crossing a threshold beyond manageable) as of April 1, 2020, which was used as a proxy of COVID-19 severity at the program level [18].

Kruskal-Wallis rank sum test was used to examine changes in well-being from the baseline survey to the post survey for continuous variables. Fisher’s exact tests were used for categorical variables. The normality of distribution for continuous outcomes was checked and linear regression models were used to examine the association between continuous outcomes and predictors and to control for the baseline confounding variables. The coefficient estimate βs and corresponding p-values and 95% confidence intervals (95%CI) were reported. The proportional odds assumptions were checked, and proportional odds models were used for ordinal outcomes. Observations with missing values were excluded from the related analysis. A p-value less than 0.05 was considered statistically significant. R version 3.6.3 was used for all the analyses (R Core Team, 2020; R Foundation for Statistical Computing, Vienna, Austria).

## Results

In total, 144 (65%) residents responded to the baseline survey, 73 (33%) responded to the post survey, and 49 (22%) completed both surveys. Among the 49 participants who completed both surveys, seven were from the four Stage one residency programs, 34 were from the eight Stage two residency programs, and eight were from the three Stage three residency programs. Table 1 summarizes the numbers of programs and participants by COVID-19 severity designation. Responders from programs of higher COVID-19 severity had lower response rates after the start of the pandemic (β = -0.11, 95%CI: -0.23, 0.01), but the COVID-19 severity effect was not statistically significant (P = 0.06).

**Table 1:** Numbers and Percentages of Programs and Participants by ACGME COVID-19 Stage Designation Stage One = business as usual, Stage Two = increased but manageable clinical demand, Stage Three = clinical demand crossing a threshold beyond manageable, Baseline Survey = measures collected in September and October 2019, Post Survey = measures collected during the first COVID-19 surge ACGME: Accreditation Council for Graduate Medical Education; COVID-19: coronavirus disease 2019

	Stage One	Stage Two	Stage Three	Total
Residency programs	4 (27%)	8 (53%)	3 (20%)	15 (100%)
Number of residents	33 (15%)	127 (57%)	62 (28%)	222 (100%)
Responded to only the baseline survey	23 (16%)	81 (56%)	40 (28%)	144 (100%)
Responded to only the post survey	11 (15%)	48 (66%)	14 (19%)	73 (100%)
Completed both surveys	7 (14%)	34 (69%)	8 (16%)	49 (100%)

We did not find a statistically significant difference in any of the well-being outcomes between the fall of 2019 and the spring of 2020 after the initial outbreak of COVID-19 among participants who completed both surveys (Table 2).

**Table 2:** The well-being status at baseline survey versus post survey ^1^ Kruskal-Wallis rank sum test; ^2 ^Fisher’s Exact Test for Count Data SD = standard deviation; Stress = stress level measured by the Perceived Stress Scale; pss = overall perceived social support measured by MSPSS. The items of MSPSS were further divided into factor groups relating to the source of social support, namely family (fam), friends (fri), or significant other (so); Miniz = burnout measured by 15-item Mini-Z, with a total score >=60 considered a positive learning environment; Minizq1 = the single-item satisfaction score; Minizq2 = the single-item burnout score; phq9 = total score of degree of depression severity as measured by PHQ-9; phq9_cate = categorization based on the total score (No depression=0, Minimal depression=1-4, Mild depression=5-9, Moderate depression=10-14, Moderately severe depression=15-19, Severe depression=20-27); Who = well-being measured by the WHO-5 Well-Being Index

	Baseline Survey (N=49)	Post Survey (N=49)	p-value
Stress			0.856^1^
Mean (SD)	23.673 (7.409)	23.102 (7.600)	
pss			0.134^1^
Mean (SD)	69.000 (10.118)	70.857 (11.986)	
pss_so			0.279^1^
Mean (SD)	23.796 (5.144)	24.592 (5.311)	
pss_fam			0.675^1^
Mean (SD)	23.041 (4.756)	23.000 (5.496)	
pss_fri			0.308^1^
Mean (SD)	22.163 (4.598)	23.265 (3.598)	
Miniz			0.268^2^
Positive	2 (4.1%)	6 (12.2%)	
Negative	47 (95.9%)	43 (87.8%)	
Minizq1 (satisfaction)			0.515^1^
Median	4	4	
Q1	3	4	
Q3	5	4	
Minizq2 (burnout)			0.397^1^
Median	3	4	
Q1	3	3	
Q3	4	4	
phq9			0.558^1^
Mean (SD)	5.327 (4.185)	4.980 (4.166)	
phq9_cate			0.719^2^
No depression	4 (8.2%)	5 (10.2%)	
Minimal depression	20 (40.8%)	20 (40.8%)	
Mild depression	20 (40.8%)	16 (32.7%)	
Moderate depression	3 (6.1%)	7 (14.3%)	
Moderately severe depression	1 (2.0%)	1 (2.0%)	
Severe depression	1 (2.0%)	0 (0.0%)	
Who			0.356^1^
Mean (SD)	13.020 (4.356)	13.837 (4.497)	

While we did not find a strong association between the COVID-19 severity at the residency program level and most well-being measures, respondents from programs of higher COVID-19 severity had lower WHO-5 scores (β = -2.53, 95%CI: -4.60, -0.47, P = 0.02) at post survey on average (Stage one: 16.22±3.60, Stage two: 14.06±4.47, Stage three: 11.13±4.05). However, after controlling for baseline survey WHO-5 scores, the difference was not significant (β = -1.14, 95%CI: -3.04, 0.77, P = 0.24). No difference was identified between programs of varying COVID-19 burden for reported perceived stress, social support, or burnout, with or without controlling for baseline survey scores. The odds of having a more severe depression score were 3.89 (95%CI: 0.63, 24.87) times higher in Stage three than in Stage one programs at the post survey. The difference is notable but not statistically significant (Stage one: 3.56±2.19, Stage two: 4.97±4.27, Stage three: 6.25±4.80, p = 0.144). After controlling for baseline survey depression severity, the odds decreased to 1.75 (95%CI: 0.18, 16.97, P= 0.62).

**Figure 1:**
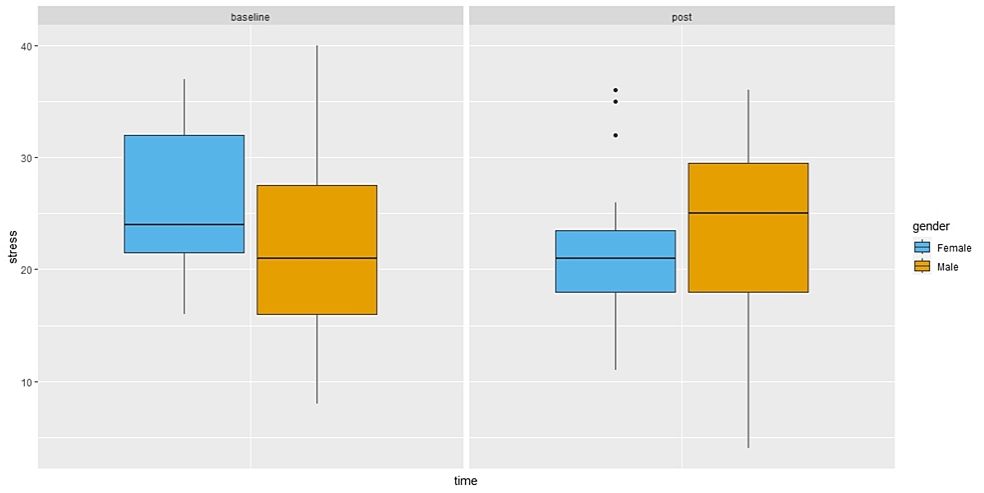
Perceived stress by time and gender. Boxplots showing median (central horizontal line), 25th percentile (lower end of the box), and 75th percentile (upper end of the box) for perceived stress by Time (baseline survey versus post survey) and Gender (female versus male). The upper whisker represents scores larger than 75th percentile but less than 1.5 times the upper quartile. The lower whisker represents scores less than 25th percentile but greater than 1.5 times the lower quartile. The dots represent those outliers that are greater (or less) than 1.5 times the upper (or lower) quartile.

**Figure 2:**
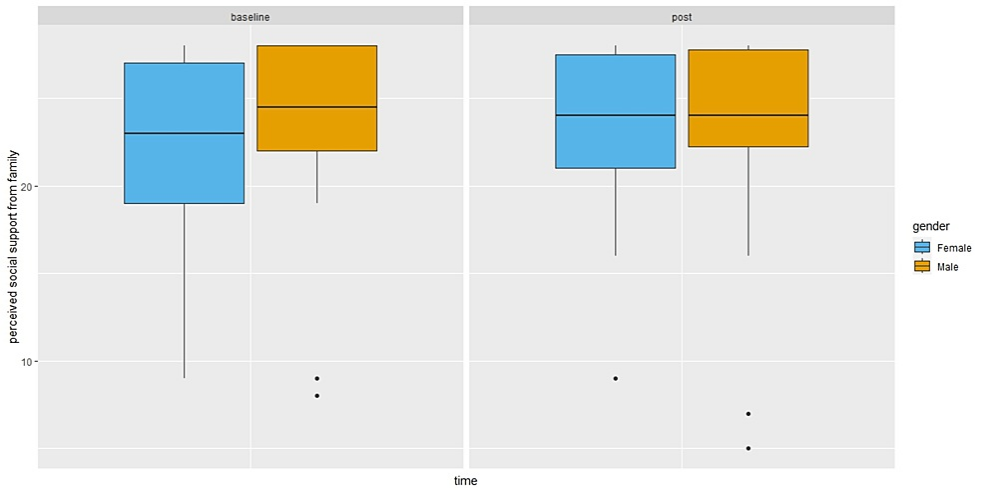
Perceived social support from family by time and gender. Boxplots showing median (central horizontal line), 25th percentile (lower end of the box), and 75th percentile (upper end of the box) for perceived social support from family by Time (baseline survey versus post survey) and Gender (female versus male). The upper whisker represents scores larger than 75th percentile but less than 1.5 times the upper quartile. The lower whisker represents scores less than 25th percentile but greater than 1.5 times the lower quartile. The dots represent those outliers that are greater (or less) than 1.5 times the upper (or lower) quartile.

Male participants reported lower friend support than females in the post survey, without controlling for baseline survey perceived friend support (β = -2.63, 95%CI: -4.53, -0.74, P = 0.01). However, the gender difference was no longer significant after controlling for baseline survey values. As shown in Figure 1, male participants reported higher stress (β = 4.05, 95%CI: 0.42, 7.67, P = 0.03) in the post survey compared to females after controlling for baseline scores. They also reported lower social support from family (β = -6.57, 95%CI: -11.64, -1.51, P = 0.01) in the post survey compared to females after controlling for baseline scores (Figure 2).

**Figure 3:**
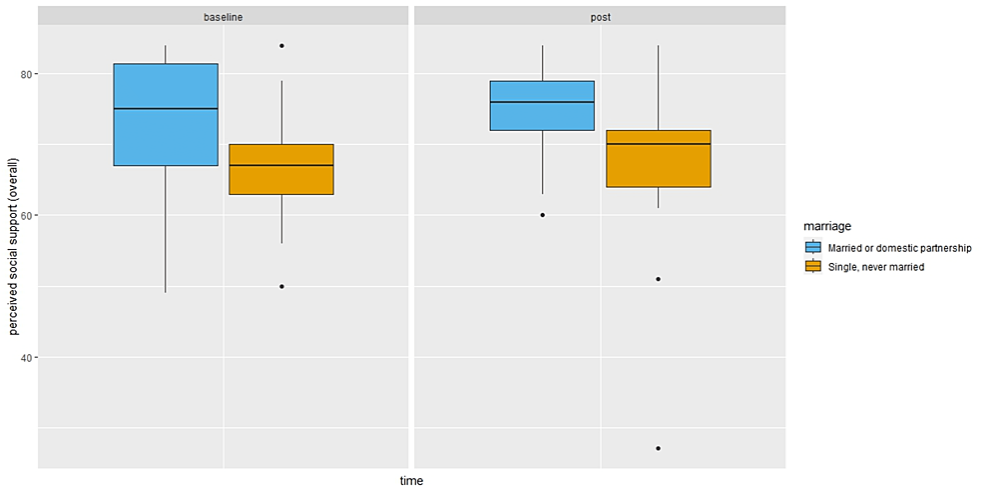
Perceived overall social support by time and marital status. Boxplots showing median (central horizontal line), 25th percentile (lower end of the box), and 75th percentile (upper end of the box) for perceived overall social support by Time (baseline survey versus post survey) and Marital Status (married or domestic partnership versus single or never married). The upper whisker represents scores larger than 75th percentile but less than 1.5 times the upper quartile. The lower whisker represents scores less than 25th percentile but greater than 1.5 times the lower quartile. The dots represent those outliers that are greater (or less) than 1.5 times the upper (or lower) quartile.

**Figure 4:**
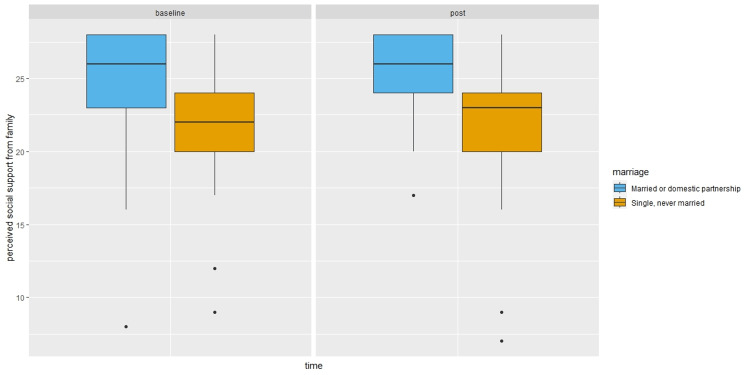
Perceived social support from family by time and marital status. Boxplots showing median (central horizontal line), 25th percentile (lower end of the box), and 75th percentile (upper end of the box) for perceived social support from family by Time (baseline survey versus post survey) and Marital Status (married or domestic partnership versus single or never married). The upper whisker represents scores larger than 75th percentile but less than 1.5 times the upper quartile. The lower whisker represents scores less than 25th percentile but greater than 1.5 times the lower quartile. The dots represent those outliers that are greater (or less) than 1.5 times the upper (or lower) quartile.

Compared to single or separated participants, married participants or those with domestic partners reported higher social support in the post survey (β = 5.79, 95%CI: -0.65, 12.23, P = 0.03), although the magnitude of the difference decreased after controlling for baseline survey perceived social support (β = 2.13, 95%CI: -3.50, 7.77, P = 0.35) as shown in Figure 3. In particular, married participants or those with domestic partners reported higher family support in the post survey than single or separated residents (β = 3.15, 95%CI: 0.13, 6.17, P = 0.01). Again, the difference was no longer statistically significant at the 0.05 level after controlling for the baseline survey perceived family support (β = 1.67, 95%CI: -0.77, 4.11, P = 0.10) as shown in Figure 4. Finally, the odds of having more severe depression among married/domestic partnership participants were 1.41 times (95%CI: 0.51, 3.94, P = 0.51) that of single/separated participants, which increased to 3.08 times (95%CI: 0.82, 12.83, P = 0.11) after controlling for baseline depression severity.

## Discussion

Statement of principal findings

In this longitudinal survey-based study, we aimed to assess the impact of the early COVID-19 pandemic on the well-being of anesthesiology residents in the US. Surprisingly, there were no statistically significant differences in any of the well-being outcomes between pre-pandemic and the beginning of the pandemic. Equally surprising, there were no statistically significant differences relative to the impact of the pandemic at a given institution based on disease burden.

Interpretation within the context of the wider literature

There are several potential reasons for these results that may be correlated to proactive supportive initiatives put in place by residency and graduate medical education leadership. When the pandemic began, residency programs around the country employed various strategies to protect their residents and minimize their exposure to COVID-19 (e.g., intubations of COVID-19 patients done by attendings only). At this same time, elective surgeries were canceled nationwide, leading to lower OR workloads for a large proportion of residents. Despite a decrease in workload, resident pay and benefits were not affected (even though some faculty members were furloughed). Also, the ACGME mandated that time off from clinical duties due to COVID-19 illness or quarantine would not be counted as sick time. In addition, the American Board of Anesthesiology Basic Examination was postponed, which may have specifically decreased the stress level for these CA-1 residents. It is also possible that we were in the “honeymoon phase” of disaster at the time of our post survey, in which there was plentiful support and community building for healthcare workers deemed as heroes at that time [19]. Similar results were found in a study of vascular surgery trainees during the first COVID-19 surge, with their trainees reporting low levels of anxiety and utilization of healthy coping strategies [20]. Interestingly, a separate study comparing perceived stress, anxiety, and depression during COVID-19 found that healthcare workers had better well-being than those in other professions [21].

When comparing well-being differences by gender, we found that male residents reported higher stress levels and decreased levels of family support compared to female residents, which was sustained when controlled for baseline perceived stress. This finding suggests male residents may be a vulnerable population in this setting and may warrant additional program support, especially since males are known to be more successful in suicide attempts [22,23]. Interestingly, Yan et al. assessed gender differences in stress responses to the pandemic in the Chinese population and found that the female gender had increased levels of stress, although frequent contact with colleagues was found to be protective [24]. A recent survey of the general population by the American Psychological Association, over a period of four years, found that 28% of women had a stress level of > 8 on a 10-point scale compared to 20% of men [25]. The survey also highlighted that 49% of women reported their stress level increased over five years compared to 39% of men [25]. As women have been shown to have higher levels of stress in other literature, the results we observed may be attributed to lower levels of support experienced by male residents. It is also possible that non-response bias plays a role in the finding that the most burned-out female residents did not respond. Nonetheless, it is difficult to speculate the source of these apparent gender differences, and we hope to explore this further in future studies.

Married residents, or those with domestic partners, reported higher levels of family support compared to single or separated residents, although this finding was no longer significant once controlled for baseline perceived family support. The pandemic proved to be very socially isolating for many people. Perhaps living with a significant other helped to maintain the human connection during this otherwise isolating time.

Strengths and limitations

To our knowledge, this is the first multi-site study that assessed the impact of the COVID-19 pandemic on the well-being of anesthesiology residents in the US using a repeated survey design. However, this study had several limitations. First, a higher response rate would have strengthened the results. Without all residents completing the survey, we do not know if there are any response patterns that could bias the results. Interestingly, individual response rates per residency program for the post survey were not significantly different based on the severity of the program’s COVID-19 burden, indicating that the severity of the pandemic did not impact the willingness to engage with the study. Therefore, the stability of the well-being measures held true regardless of the severity of the pandemic. Nonetheless, the relative paucity of Stage one and Stage three programs at the time when the study was conducted may have limited the generalizability of the study findings. Second, the effective total sample size is small, which may limit the power of the study to detect significant differences. However, a posthoc power analysis showed that, with a significance criterion of α = .05 and power = .80, the minimum sample size needed is n=1362 in order to detect a perceived stress level difference between the baseline survey and the post survey, which suggests a trivial between-survey stress difference. Third, the scales used to assess well-being may not have been sensitive enough to detect differences. To mitigate this limitation, we used five validated scales to assess various aspects of well-being, which should have decreased the likelihood of false detection. However, these surveys are meant to assess well-being and not the impact of an event. Additionally, the data collected was not initially intended for the assessment of well-being changes during a pandemic, but rather for a separate longitudinal study regarding overall well-being and social support interventions. Although the post survey coincided with the first COVID-19 surge, this may not have captured the full picture of the stressors or events unfolding at a given residency program or to a given resident at that point in time. It is possible that the well-being scores may have shifted if the assessment had been repeated after surgical volumes returned to pre-pandemic levels, to coincide with the future COVID-19 surges, or simply as the stress of the pandemic’s impact continued over time. Finally, data were collected early in a longstanding pandemic. At this time, the pandemic was a shock to many people with various unknowns. Although conclusions regarding the overall impact of the COVID-19 pandemic on anesthesiology residents cannot be drawn, the initial responses and feelings from residents during this study period may parallel those felt during personal or global crises that our trainees may face.

Implications for policy, practice, and research

In contrast to our hypothesis, the COVID-19 disease burden at an anesthesiology residency program did not appear to significantly impact well-being scores compared to pre-pandemic levels based on our survey data. Residents most vulnerable to the impact of the COVID-19 pandemic on well-being appeared to be male and single participants. As a result, targeted well-being interventions, including those aiming to increase social support, for higher-risk resident groups may be indicated while making sure not to do this at the expense or to the detriment of other groups. Future research is warranted to provide insight into how this longstanding pandemic has affected residents’ work life and wellness, with particular attention to the dynamic nature of pandemic severity.

## Conclusions

The local COVID-19 severity at a residency program did not appear to disproportionately impact well-being scores among US PGY2 anesthesiology residents. Those most vulnerable to diminished well-being appeared to be male and single participants. Findings from this multi-site repeated survey study should be interpreted with caution and in combination with evidence from other relevant studies due to the low response rate. Future work is needed to assess the longstanding COVID-19 pandemic on resident well-being.
